# A Principle-Based Approach to Visual Identification Systems for Hospitalized People with Dementia

**DOI:** 10.1007/s11673-023-10315-x

**Published:** 2023-11-29

**Authors:** T. V. Brigden, C. Mitchell, K. Kuberska, A. Hall

**Affiliations:** 1 PHG Foundation, University of Cambridge, 2 Worts’ Causeway, Cambridge, CB1 8RN England; 2 The Healthcare Improvement Studies (THIS) Institute, University of Cambridge, 2 Worts’ Causeway, Cambridge, CB1 8RN England

**Keywords:** Dementia, Ageing, Hospital care, Visual identification systems, Ethical principles, United Kingdom

## Abstract

A large proportion of hospital inpatients are affected by cognitive impairment, posing challenges in the provision of their care in busy, fast-paced acute wards. Signs and symbols, known as visual identifiers, are employed in many U.K. hospitals with the intention of helping healthcare professionals identify and respond to the needs of these patients. Although widely considered useful, these tools are used inconsistently, have not been subject to full evaluation, and attract criticism for acting as a shorthand for a routinized response. In order for visual identifiers to be used effectively in acute care settings, thorough consideration must be given to the ethical and legal issues that are engaged in this context, and their potential benefits and harms must be weighed and balanced. This paper proposes a set of legal and ethical principles that can be used to guide the implementation of visual identifiers. Together, these principles provide a framework applicable in the design and implementation phases to systematically identify relevant considerations arising from the use of these tools. We outline some tensions that arise between principles and conclude that selecting a preferred moral framework could help to guide decision-making, as does clarity around the purpose and objectives of the identifier.

## Introduction

Visual identifiers have been implemented in many hospitals in the United Kingdom (U.K.) to improve the care of those affected by cognitive impairment. Despite significant appetite for these tools and their potential positive impact, they pose risks to patients that need to be considered and mitigated against. In the absence of a standardized visual identification system for people with dementia, different schemes have been implemented in the United Kingdom in an unsystematic manner, ranging from established national schemes to locally developed ones. Additionally, there has been no thorough evaluation of the ethico-legal considerations that might be generated by their use. In order to explore the use of these tools in U.K. hospital settings, the DA VINCI (Developing A Visual IdeNtification method for people with Cognitive Impairment in institutional settings) project was undertaken; a multi-disciplinary research programme involving four linked studies. In this paper, we propose a set of ethical and legal principles that are engaged in the context of visual identifiers, and which can be used to systematically and purposely evaluate the accompanying benefits and risks. Our aim was to consider the implications of these principles for the use of visual identifiers within a single jurisdiction, taking into account the legal framework and policy landscape in the United Kingdom. In doing so, we also aimed to make explicit the potential for trade-offs between the principles, some examples of which are outlined in this paper. We conclude that these have to be weighed keeping in mind the context and purpose of the identifier, and that person-centred care might provide a useful indicative framework to prioritize the principles and support responsible policy development.

## Visual Identifiers in Dementia Care

Approximately a quarter of acute care hospital beds in the United Kingdom are occupied by patients living with dementia (Royal College of Psychiatrists 2019). Providing high quality person-centred care to those with dementia and other forms of cognitive impairment is essential but challenging, as these patients may have particular needs that are not immediately recognizable to the staff responsible for their care. These can relate to communicating their preferences and assistance with nutrition and/or other basic activities. Where the provision of care fails to cater for their needs, people with dementia may suffer harm and distress, such as falls, immobility, incontinence, and functional decline (Hermann, Muck, and Nehen 2015). This is why the Royal College of Psychiatrists recommends that hospitals have special systems in place to care for this patient group appropriately (Royal College of Psychiatrists 2019).

One way to improve the quality of care of people with dementia is to introduce visual identifiers—signs and symbols attached above patients’ beds, on their person, or in their notes to direct attention to particular aspects of patient care. These tools can be used to indicate the presence of a variety of different risks or considerations, including visual impairment, hearing loss, and falls risk. In the context of caring for people with dementia, they aim to indicate a dementia diagnosis (or cognitive impairment more broadly) to those involved in patient care, so that they might better respond to the needs of patients with dementia. In addition to the heterogeneity around the purpose and scope of identifiers, a range of different terms are also used, such as “Cognitive Impairment Identifier” (Yates, Theobald, and Morvell 2009; Murray, et al. 2019); in this paper we refer to “visual identifiers.”

There is no systematic data about the global use of visual identifiers for patients with dementia; it is, however, known that they are used in Australia and the United States for cognitive impairment and delirium more broadly. In Australia, the Dementia Care in Hospitals Program was developed in 2004 by Ballarat Health Services; the use of a bedside Cognitive Impairment Identifier is one component of this programme (MacDermott, et al. 2017). It has since been implemented in a number of hospitals across four Australian states, and other similar programmes have been introduced with modification to suit the local context (Fox et al. 2023).

In the United Kingdom, the 2019 Royal College of Psychiatrists National Audit of Dementia Care reports that 93 per cent of general hospitals in England and Wales use a visual identification system of some description in their wards (Royal College of Psychiatrists 2019). These identifiers can vary across hospitals and can take the form of wristbands, patient profile documents, symbols, or notices placed above a patient’s bed and/or in their patient notes, or notifications in digital systems, for example. However, the presence of visual identification schemes in hospitals does not guarantee that they are used consistently or have been subject to formal evaluation.

There are a variety of identifiers in use; each has slightly different purposes, ranging from increased efficiency to promoting person-centred practice. Some visual identifiers are part of established national schemes and others locally developed. Some act as standalone identifiers of cognitive impairment, and others—such as the Butterfly Scheme—use visual identifiers as components in a wider care approach (Kuberska, et al. 2022).

These technologies were introduced with the intention of enabling good quality care for people with dementia, but inconsistent, improper, or unreflective use may undermine their objectives. Evidence suggests that these technologies can sometimes lead to less personalized care and become a shorthand for a routinized response or can quickly become invisible and blend into an ocean of signage, posters, and notices, medical records, and forms that proliferate in the ward (Featherstone, Boddington, and Northcott 2020). The implementation of similar tools for multiple applications may also contribute to an abundance of competing wristbands or signage above patients’ beds and potentially lead to confusions or dilute the impact of the dementia visual identifier.

There is, however, an appetite for their use, as shown by a recent survey of practice in U.K. hospitals which reported that staff responses were largely positive about key functions of the identifiers (Kuberska, et al. 2022). The authors also found that those in hospitals that do not currently use visual identifiers tended to be enthusiastic about their potential. Respondents identified some advantages, such as supporting staff to tailor their approach to the patient, especially those who are not a regular part of the care team. On the other hand, they also raised concerns about staff making assumptions about the homogeneity of needs of people with identifiers, as well as the ethical and legal implications of unclear consent processes.

Currently, there is no overarching framework for considering how a visual identification system should be developed and implemented. In this paper we propose a set of ethically and legally grounded principles that can be used to guide the implementation of these systems for the benefit of patients with dementia.

## Methods

As part of a wider programme of studies examining the use and design of visual identification systems for people with dementia (The Healthcare Improvement Studies Institute 2023), we conducted a comprehensive analysis of the key ethical and legal/regulatory issues arising from the current and potential use of visual identifiers (Brigden, et al. 2020). The overall objective of the DA VINCI programme is to better understand the use of visual identification for individuals with suspected or diagnosed dementia in hospital settings. This research programme comprised four linked studies carried out by teams from different organizations and institutions. These included: a survey of current practice around the visual identification of people with cognitive impairment in hospital settings (Phase 1a); a desk-based analysis of the ethical and legal issues involved in the use of such systems (Phase 1b) ; a qualitative study, including in-depth case studies of and interviews about current applications of visual identifiers in hospitals with staff, people with dementia and their carers (Phase 1c); and a co-design study comprising a series of participatory workshops with carers and staff to co-develop a set of design principles (Phase 2a). In Phase 1b, the analysis of ethical and legal issues, we aimed to provide a theoretical framework for subsequent phases.

We identified relevant ethical and legal considerations through desk-based research, drawing on peer reviewed literature, grey literature, and official publications. Computerized searches were carried out using Pubmed, SCOPUS, Medline, CINAHL, and ProQuest (APA PsychInfo (1806 – current) and British Nursing Index (1994 – current)), in order to identify academic literature discussing ethical considerations that arise as a result of using visual identifiers for dementia. Due to the limited literature available on visual identifiers specifically, the inclusion criteria were broadened to identify relevant analogous literature. The search strategy used covered the following search terms: dementia OR “cognitive impairment” OR Alzheimer* AND emblem* OR symbol* OR wristband* OR label* OR butterfly OR forget-me-not OR “visual identif*” OR “patient identif*” OR bracelet* AND stigma OR dignity OR ethic* OR attitud* OR privacy OR moral* OR autonomy. This was supplemented with additional targeted searches and snowballing to identify additional relevant literature (Brigden, et al. 2020, 78).

Common themes, tensions, and challenges identified in the ethical and legal analyses were then used to develop a set of key principles that are engaged in this context, to inform the development and implementation of visual identifiers going forward. The research process involved a team of researchers working in a multidisciplinary manner across legal, ethical ,and social sciences literatures. TB is a bioethicist and policy analyst whose research focuses on ethical issues arising from biomedical innovation and personalized healthcare, informed by her work on NHS ethics committees. AH led the legal and ethical analysis comprising Phase 1b of the DA VINCI project: her legal and ethical research on regulation and ethical issues arising from implementing novel biomedical technologies is informed by work in developing professional best practice guidance for clinical professionals and her former work as a practicing nurse and lawyer. CM is a specialist in health law and policy, with experience of qualitative research and normative research in law and ethics. He carried out legal analysis as part of this project. KK is a medical anthropologist with research experience in socio-legal studies, bioethics, and healthcare improvement studies; she was the research lead on the DA VINCI project.

We consolidated and tested these draft principles with an Expert Collaborative Group of 20 people, who had been selected to offer guidance throughout the phases of the DA VINCI project (The Healthcare Improvement Studies Institute 2023). This group included hospital staff, patient and carer representatives, individuals who had led the development of existing identification and dementia care systems, clinical and non-clinical academic experts in related fields, third-sector organizations, and collaborators in the wider study. Their feedback and insights were then used to further refine the principles.

In this paper we build on the principles to identify key questions and considerations in the design and implementation of visual identification systems, explore some examples of tensions that can arise, and discuss how the principles might be balanced.

## Key Principles and Considerations to Guide the Use of Visual Identification Systems

**Fig. 1:**
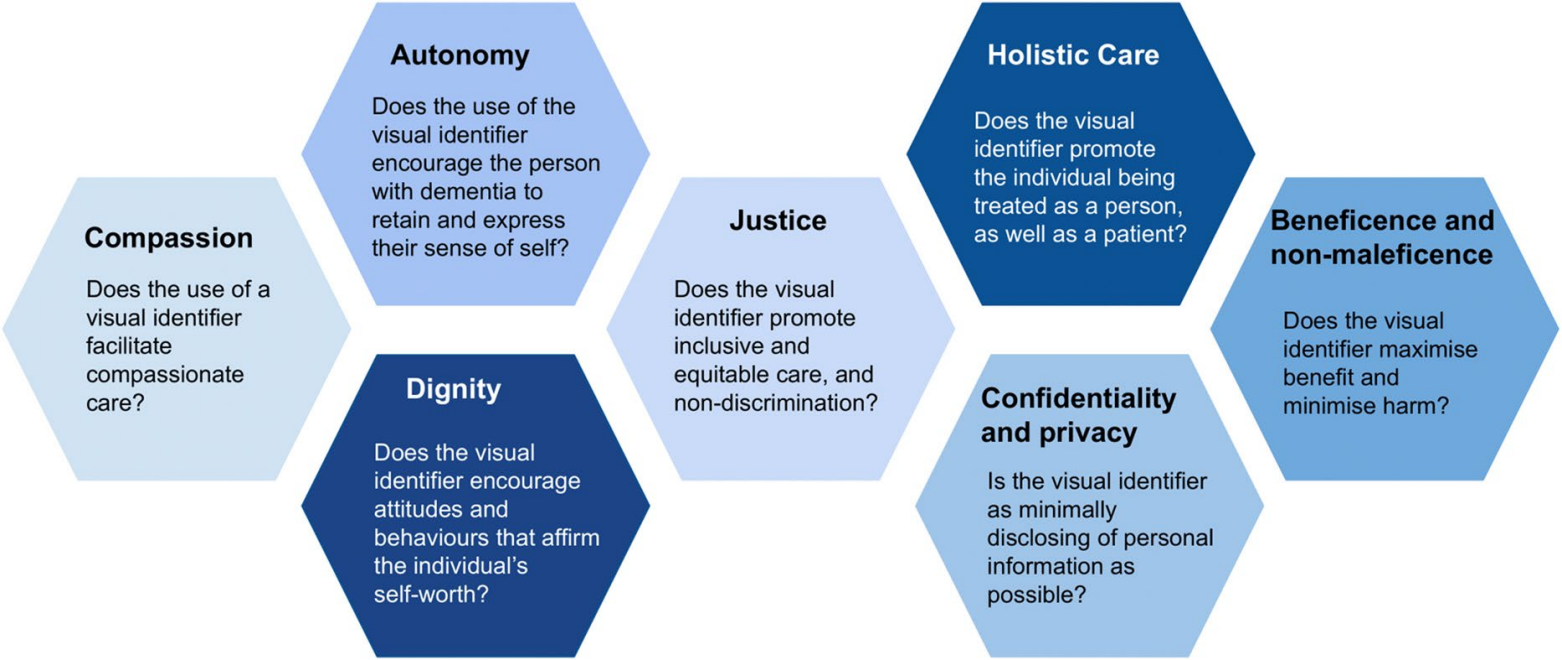
Key principles for the use of visual identifiers in the care of people with dementia and the corresponding questions that they raise

The principles that we identified were informed by ethical and legal literature pertaining to the challenges that individuals with dementia face in their care. These key principles are autonomy; beneficence and non-maleficence; dignity; justice; confidentiality, privacy and the protection of personal data; compassion; and holistic care (Brigden, et al. 2020). Most of these are established bioethical principles, but they are supplemented by principles derived from ethical and legal frameworks relevant to care for people with dementia such as the ethics of care (Maio 2018), virtue ethics (Harding 2017), and person-centred healthcare (Coulter and Oldham 2016). These principles provide an analytical framework through which the considerations and implications of visual identifiers can be identified and assessed systematically. They enable a contextual assessment of a proposed visual identification system or tool, shedding light on the particular principles that might be engaged and some of the issues that could arise. Most importantly, consideration of these principles in the context of caring for people with dementia gives rise to specific questions for practitioners or policymakers considering the design or implementation of a visual identifier (Fig. 1).

## Does the Use of the Visual Identifier Encourage the Person with Dementia to Retain and Express Their Sense of Self?

Autonomy is viewed as a fundamental principle underpinning human rights and is also one of four *prima facie* binding moral norms commonly thought to guide practical decision-making in bioethics (Beauchamp and Childress 2001). Identifying tools to promote autonomy is particularly important in the context of older people and those with dementia, who can more easily become disempowered in healthcare settings (McWilliam 1994). It is widely recognized that conceptions of autonomy that solely value rationality, independence, and capacity are untenable in the context of caring for people with dementia, particularly in a hospital setting (McCormack 2001). As a result, it is particularly important to adopt a more nuanced interpretation of autonomy, consistent with the concept of interrelationship and a degree of dependency upon others which may sometimes characterize interactions with people with dementia (Perkins, et al. 2012; Wolfe, et al. 2021; Kitwood and Bredin 1992). Research exploring what autonomy means to those with a diagnosis of dementia and family carers has demonstrated a range of perspectives, with some valuing retaining independence and self-expression, others accepting dependence but wishing to be included, and others placing less emphasis on this and more on the need for opportunities for connection with others (Wolfe, et al. 2021). These findings indicate that a one-size-fits-all approach to thinking about and supporting matters of autonomy is of limited utility for people with dementia.

Rather than solely identifying a condition, visual identifiers must be used as a first step in a care approach where the care provider acts as an advocate for the person with dementia or suspected dementia, helping to foster their unique capabilities, skills, relationships, and preferences which are necessary for self-expression and the promotion of personal autonomy. In this way the healthcare professional can help encourage individuals to retain and express their sense of self, both through medical decision-making and more widely.

If implemented ineffectively, however, visual identifiers might undermine individual autonomy through drawing attention to the label of dementia and fostering a homogenized understanding of patient needs (for example, that such a patient must be unable to walk independently or in need of high levels of support at mealtimes) (Featherstone, Boddington and Northcott 2020).

## Does the Visual Identifier Encourage Attitudes and Behaviours That Support the Individual’s Self-Worth?

Literature suggests that, much like autonomy, the dignity of older people is often undermined in healthcare settings (Lothian and Philp 2001). Basic human dignity is considered to be intrinsic to all humans, regardless of faculties, acting as a ground for human rights (Gewirth 1992). Personal dignity, however, is a type of dignity that is subjectively experienced by an individual and relates to a sense of worthiness. It can be influenced both by an internal aspect (the worth and self-respect an individual ascribes to themself) and by an external aspect (the worth and value ascribed by others) (van Gennip, et al. 2016). Those with dementia are particularly at risk of suffering a loss of personal dignity as the result of the direct impact the disease may have on their physical and mental capabilities, identity, and sense of self.

Personal dignity has different meanings to different people and is dependent to some extent upon cultural and societal norms. There has been much debate about how to achieve dignified care with emphasis being placed on aspects such as compassionate care, genuine respect for the person, person-centred environments, and maintaining the individual’s sense of meaning and purpose (Tranvåg, Petersen, and Nåden 2013). It is crucial to consider whether the visual identifier enables attitudes, behaviours, and practices that affirm the individual’s self-worth, as human interactions have the potential to be “dignity encounters” (Jacobson 2009) that can either preserve or undermine dignity. For example, studies have shown that feeling respected, listened to, taken seriously, treated with kindliness, given health-related information in a gentle manner, and adopting positive realism, are among the most crucial dignity-preserving qualities found in healthcare interactions (Tranvåg, Petersen, and Nåden 2014). This is congruent with the use of visual identifiers, which can open up a dialogue with the patient. The perceived priorities of the system also impact dignity experience. Having similar status and rights as other patient groups was identified by Tranvåg, et al. as a fundamental quality crucial for preserving dignity experience (Tranvåg, Petersen, and Nåden 2014).

## Does the Use of a Visual Identifier Facilitate Compassionate Care?

Compassionate care can promote staff/patient interactions that support personal dignity. Following this principle, interactions with healthcare professionals should be underpinned by compassion and meaningful engagement, rather than other priorities (such as administrative expediency). In doing so, the implementation and delivery of the visual identifier should reflect the compassion that motivates its use.

Compassion is not a traditional ethical principle but instead a character trait or emotion that is widely considered to be a key component of quality care for people with dementia. Virtue ethics (a theory of morality grounded in character-centred moral judgements) calls for individuals to be compassionate, arguing that feeling compassion regulates action. In other words, the compassionate person will not only feel compassion but act compassionately (Crisp 2008). According to virtue ethics, it is not sufficient for the visual identifier to be beneficial, but it must be used in the right way and for the right reasons if it is to be virtuous. Although aspects of this seem aspirational, viewing compassion as a virtue could offer lessons for the use of visual identification systems, providing insight into the assessment of individual patient needs and ways to address these. In the context of caring for people with dementia, compassion could usefully inform what matters to the patient, e.g. where the patient’s wishes must be constructed through making a substituted judgement (Mental Capacity Act 2005, s4(6)).

Embedding compassion within visual identification systems may be even more demanding, requiring that they are a facet of a compassionate system enacted by compassionate healthcare professionals. The environment, medical culture, and organizational structure of the hospital are key to facilitating compassionate care, as is the education and training of the healthcare professional to enable them to identify, understand, and respond to care needs. Some of the practical challenges in achieving this are discussed later in this paper.

## Does the Visual Identifier Promote Inclusive and Equitable Care and Non-Discrimination?

The principle of justice requires that equals be treated equally and unequals unequally (Aristotle (Ross trans.) 2009). In its contemporary form, this principle is sometimes expressed as follows: “Individuals should be treated the same, unless they differ in ways that are morally relevant to the situation in which they are involved.” Disabilities, such as dementia, raise issues of justice as people with dementia may suffer from both social injustice (relating to treatment by others) and distributive injustice (relating to the allocation of resources). For example, they may face disadvantages and stigmatization as a result of a societal bias and poor access to required adjustments.

Equity aims to promote justice, through calling for everyone to be treated according to their circumstances. In the context of healthcare, this will require altering approaches to care in light of relevant patient information and may in some instances require directing additional resources to people with dementia. The visual identifier can act as a tool to enable equitable treatment, through making the patient’s otherwise hidden needs plain to those charged with their care. It should be noted, however, that when implemented without an appropriate care approach and sufficient training on the variable aetiology of dementia, the identifier also has the potential to promote generalized constructions of “the dementia patient,” facilitate stigmatization, and disenfranchise individuals with dementia.

Discrimination is a form of injustice, and therefore non-discrimination also forms a core part of the human rights framework, for example, Article 14 of the European Convention on Human Rights enshrines the right not to be discriminated against on the grounds of disability, medical condition, or genetic feature (amongst others). In the United Kingdom a variety of further laws (including the Equality Act 2010) establish more specific rights and corresponding duties on public authorities such as NHS trusts, for example, to minimize disadvantages, tailor care, and meet the needs of patients. In the context of visual identification systems, this may include considerations such as ensuring that supporting documents and processes accompanying the identifier account for those unable to understand the offer presented in the dominant/official language(s) and formats in each healthcare facility. Additionally, it will also require that individuals do not feel coerced into consenting, and that those who opt-out of using a visual identifier are not negatively impacted by this choice as far as is possible. In practice, it will be challenging to ensure that patients with and without an identifier receive the same standard of care, whilst recognizing that the provision of choice may itself be regarded as beneficial.

## Does the Visual Identifier Promote the Individual Being Treated as a Person, as Well as a Patient?

Policy initiatives, policymakers, and healthcare professionals affirm the importance of creating caring environments and cultures that are safe, holistic, and person-centred (NHS 2019; The Health Policy Partnership 2015). A holistic approach to caring for people with dementia involves seeing the individual as a unique and whole person. Rather than solely focussing on disease or symptoms, this approach emphasizes that healthcare professionals should take into account other dimensions affecting individual well-being, such as abilities, interests, needs, and preferences. The importance of hospital staff having “life-story information” has been recognized (Røsvik and Rokstad 2020). Although some visual identifiers (such as the “This is Me” patient profile document) are intended to be tools to enable this, they do, by nature, bring a diagnosis and its associated symptoms to the fore, highlighting the physical and medical dementia-related needs of a patient. In doing so, they may inadvertently contribute to erasure of the person and any other relevant medical or non-medical needs they may have. This may be exacerbated in an acute care setting, as research has shown that for a person with dementia hospitalization can lead to an increase in behavioural and psychological symptoms of dementia (Røsvik and Rokstad 2020).

These potential unintended consequences emphasize the importance of a culture where the diverse requirements of individuals with dementia are recognized. Adopting a holistic approach may emphasize implementing supporting measures alongside an identifier, for example tailoring care according to the patient’s likes and dislikes and considering their cultural or religious needs, sleeping patterns, and interests. Such an approach recognizes the limits of the identifier which cannot act as a substitution for these conversations.

## Is the Visual Identifier as Minimally Disclosing of Personal Information as Possible?

Confidentiality, patient privacy, and the right to data protection are integral to good medical practice. These separate but related concepts are particularly important in this context where highly sensitive information about a person is disclosed on the basis of trust and confidence that it will not be misused, shared without authorization, or result in an invasion of privacy or other harms (General Medical Council 2017). A disclosure of a dementia diagnosis for the direct care of the patient is part and parcel of healthcare. However, the risk of deliberate or unintentional disclosure beyond those caring for the patient makes it challenging to establish an ethical and legal basis for wider communication.

Our analysis suggested that institutional decisions about the remit of a visual identifier (e.g. whether it is applied to those with a formal diagnosis of dementia or suspected diagnosis), and its form (e.g. a sign above a patient’s hospital bed, compared to a flag in electronic patient records), could have widespread and sometimes unintended impacts on patient privacy. There are ethical and legal imperatives to ensure that the adopted approach is as minimally disclosive of private health information as possible. It may not be feasible to achieve the care goals of the identifier without some disclosure and it is important to recognize that privacy and confidentiality are not absolute—they should be balanced with other important rights and objectives.

Within the legal framework, explicit consent is the most straightforward mode of legitimizing disclosure of personal confidential information. This does not necessarily have to match the informational standards of informed consent to treatment (Chico and Taylor 2017) but patients should be aware of the general nature of the identifier and how it may disclose their health status. The challenges with this are obvious in the context of dementia where patients may lack capacity at the relevant time to make this choice. If this is the case, in England a decision may be made in the best interest of the patient in accordance with s4(7) of the Mental Capacity Act 2005, taking into account relevant views and wishes of the patient and carers. But capacity may fluctuate and it is then potentially necessary to wait until capacity to make this decision is regained for the identifier to be used.

Implied consent may be an alternative approach but it does not apply where patients lack capacity. It also does not necessarily neatly fit with the nature of some identifiers. According to the General Medical Council (GMC), “implied consent refers to circumstances in which it would be reasonable to infer that the patient agrees to the use of the information, even though this has not been directly expressed” (General Medical Council 2017, 13b). However, implied consent is interpreted in the National Health Service (NHS) context as only legitimate for disclosures which support the direct care of the patient and which are made to other members of the direct care team (Department of Health 2003). So if an identifier may be recognized and understood by a wider group, including hospital visitors, domestic staff, or even other patients, an implied consent approach may not be legally valid.

## Does the Visual Identifier Maximize Benefit and Minimize Harm?

In caring for people with dementia, as in all healthcare, the overarching ethical principles of non-maleficence (avoiding doing harm) and beneficence (doing good) are pillars of medical practice (Beauchamp and Childress 2001). These parallel principles generate overarching moral duties to protect and promote patient safety and are considered essential for fostering trust between clinicians and patients. The former gives rise to a constant duty to avoid causing harm or injury to the patient through act or omission, whereas the latter calls for positive and direct steps to promote health. However, the reality of medical practice is that doing good almost always involves, or risks involving, some aspect of doing harm and so they must be balanced against one another, as well as against other competing interests. In addition, what constitutes harm or benefit in a particular context for a particular patient is not always clear, and to act on these principles without taking into consideration others (such as autonomy) can lead to paternalism and hinder the provision of ethical care. This is a common concern in the care of people with dementia, where individuals do not always have the capacity to make significant medical decisions.

Visual identification systems aim to promote beneficence, for example through drawing attention to the specific needs of the individual; however, there are potential harms that should be guarded against. These include concerns that labelling the individual with a dementia diagnosis could be stigmatizing (Milne 2010) or lead to the dehumanization of the patient and loss of their autonomy and privacy. Further harms (as well as opportunities for benefit) might arise, including practical side-effects, e.g. that a simple identifier becomes over-relied upon to tailor patient care, in place of a more thorough assessment of their needs and wishes. Therefore, the ethical use of visual identifiers requires careful evaluation of how to balance these possible harms against the potential benefits to patient safety and well-being. Tolerances for, and views of, what constitutes harm are integral for this assessment. A range of potential harms and benefits are raised by the questions we consider in this paper; but there may be others that arise in other contexts, based on the specific drivers for an identifier in that setting. We suggest that explicitly setting out the potential benefits and harms is an important prerequisite in both designing and choosing how to implement an identifier for hospitalized people with dementia.

## Tensions and Trade-Offs

These principles cannot be prioritized simultaneously. Furthermore, they are also not entirely independent and distinct from one another. Sometimes, these principles overlap and reinforce each other, with some aspects subsumed in others. For instance, often a course of action that is autonomy-promoting will also preserve the patient’s dignity and vice versa. Nevertheless, it is also widely accepted that these bioethical principles and their legal equivalents may conflict with each other. In this way, if we were to act only according to the principle of beneficence, this would inevitably, at some stage, conflict with, and potentially undermine, the requirement to respect autonomy. Accordingly, we should envisage situations where trade-offs will need to be made in the context of the use of visual identifiers and consider what steps could be taken to address these. Notably, we are not proposing a hierarchy among these principles. Rather than arguing for the primary moral importance of one principle over another as a means to resolve tensions, we recommend considering the trade-offs and the moral issues that arise in each case.

## The Trade-Offs in Maintaining Privacy

Our legal and ethical analyses highlighted frictions between the aims of a visual identification system, i.e. bringing attention to the specific needs of the individual, and important facets of good medical practice more broadly, such as maintaining confidentiality. Recognizing these tensions is important in order to make meaningful decisions about the character and purpose of a visual identifier within a system.

A visual identifier indicates that the individual to whom it is attached has suspected or confirmed dementia. If this identifier is only applied to a patient after a formal diagnostic process, it can take the form of a diagnostic label. Although “labelling” is often interpreted as a pejorative term, as noted by Nuffield Council on Bioethics, the provision of a label for a condition is often the first, and necessary, step for appropriate care and support to be provided (Nuffield Council on Bioethics 2009). However, the application of a visual identifier involves the disclosure of otherwise private and confidential medical information—namely, a confirmed or suspected diagnosis—to all those who recognize and understand its meaning. The more hospital staff able to interpret these symbols, the greater the reach, and therefore the potential benefits of the identifier. Simultaneously, it can infringe on the patient’s privacy.

A balancing assessment must be conducted in order to decide between limiting the recognizability of the identifier to only those professionals who are directly involved in healthcare delivery for this patient group (protecting the privacy interests of these patients), or extending the reach of the system to staff not directly involved in patient care but who may nevertheless have meaningful, if fleeting, contact with patients such as ward receptionists, cleaners, security guards and porters. Indeed, the visual identifier might have the most utility for these people who are part of the wider care network within an acute care setting. However, extending knowledge of the system to this wider group may also risk disclosure of sensitive personal data to those who are not involved in caring for the patient in any way, since some of the most common symbols—such as the butterfly or forget-me-not—may be recognizable to visitors and other patients as a symbol of dementia. Finding a proportionate approach which maximizes the potential benefits whilst minimizing privacy breaches is a considerable challenge.

Reconciling these tensions through seeking the individual’s consent or authorization may provide a practical way forward that maximizes these principles whilst recognizing that patients’ privacy, confidentiality, and rights to data protection are not absolute (General Medical Council 2017). However, relying on patient consent for use of an identifier may itself prove hard to implement consistently.

## The Challenge of Consent

Seeking consent to the use of an identifier embedded in a system of care, may offer a means of resolving the tension between preserving privacy and optimizing care described above. However, determining whether or not consent is required and which information standards apply is not straightforward, and the matter is further complicated in the context of caring for people with dementia where the patient may lack capacity to make a decision about application of an identifier. Our work suggests that a more extensive consent dialogue might be proportionate where there is less direct benefit associated with the use of the identifier, where it is implemented by staff in the absence of associated training, and where the audience for the identifier is broad.

Determining what is proportionate in a particular case may be complicated, since the benefits accorded by the tool could be lost if a disproportionately burdensome consent process is in place. Ascertaining capacity can be time-consuming, and could undo the benefit of efficiency if it takes a long time to implement on a case-by-case basis. Indeed, an online survey of staff providing care for people with dementia in acute and mental health hospitals across the United Kingdom found that in practice, consent is not always established. Kuberska and colleagues note: “Perhaps of concern, and in contradiction with the guidance supporting some schemes, the results suggested that decisions about whether to use an identifier were not always taken in discussion with patients and their informal carers” (Kuberska, et al. 2022, e12472).

While insufficient time and resources may sometimes justify omitting discussions with patients and their informal carers, such as where there is an emergency admission to a ward (Coulter and Oldham 2016), our work suggests that arriving at an agreement to use the identifier is desirable in most circumstances, even if this conversation is phrased in simple terms. These findings are confirmed by other qualitative studies on the topic (Sutton, et al. 2023).

There is another possible scenario, in which information about the visual identifier has been made available, and the patient has not objected, thereby providing an implied consent to the use of the identifier. Although this may be sufficient if the visual identifier is only recognizable as such by those providing direct care to the patient, this may not be desirable if it is designed to be recognizable outside the acute ward environment and by a wider group of individuals. An alternative interpretation of implied consent may be feasible but current NHS and GMC guidance would need to be addressed to have confidence in a different approach.

## Discussion

Visual identifiers have the potential to promote or undermine important ethical and legal principles depending on how they are developed and deployed. Although they are increasingly implemented across hospitals in the United Kingdom, there is currently little evidence on whether and how visual identifiers improve staff and patient experiences. The few conducted studies indicate that visual identification systems have considerable potential, but used ineffectively, they can undermine the benefit of the identifier, or result in patient harm. Amongst the concerns identified in these research studies are lack of staff training, unclear and inconsistent consent processes, and possible anxieties that the use of indicators may cause for patients and carers (Featherstone, Boddington, and Northcott 2020; Kuberska, et al. 2022).

The principles that we propose can be used to mitigate against some of these potential harms. However, as there is no hierarchy amongst the principles on a theoretical level—no one principle trumps another—the setting and context in which the visual identification system is embedded remains central to these decisions. In other words, in order to carry out this balancing act of risks and benefits, it is important to know what you want to achieve. This raises complexities in the context of visual identifiers as they are heterogeneous, spanning a range of purposes and users, as well as differing in regards to the information being communicated and to whom. Some types of identifier support organizational efforts to monitor and track patient outcomes, others promote efficiency on the ward, and others focus on “humanizing” patient care. Clarity around the aims and objectives of the identifier can help with the prioritization of principles, and with identifying a suitable consent process.

The principles introduce some structure into moral deliberations. However, examining the purpose for which the visual identification system is being introduced provides the situational information that can help to guide decisions about the ethical implementation of the system. For example, a visual identifier that prioritizes efficiency of care may not value autonomy and consent to the same extent as a holistic patient care approach. This may result in a symbol being applied without being supplemented by appropriate patient information, which can be time consuming for healthcare professionals to collect and revisit. It is important to note that while these purposes can conflict, they also overlap to some extent. For example, whilst efficiency can come at the cost of holistic care, inefficient care is not of benefit to the patient.

Another approach to assist ethical decision-making may be to adopt a framework, such as person-centred care, that inherently prioritizes one principle over another (Brigden, et al. n.d.). A person-centred approach means, first and foremost, that visual identifiers should not be used in isolation but rather as part of a broader care approach that employs complementary strategies to maximize their benefits and minimize potential harms. These strategies could include facilitating opportunities for recording and embedding individual beliefs and preferences through the use of advance care plans which anticipate future limitations in capacity and record priorities for ongoing care. This could also involve fully utilizing dynamic and layered approaches to consent, which take account of fluctuating capacity through repeated opportunities for engaging with the intervention and its associated benefits and harms (process consent).

Other strategies to embed person-centred care look beyond the immediate healthcare professional team. One such policy is to integrate high quality training on caring for hospitalized people with dementia and the use of identifiers into a wide range of hospital employee roles, including non-clinical staff, such as porters or cleaners. A study in Australia has shown that implementing a dementia care programme that incorporates an identifier and hospital training for clinical and non-clinical staff leads to improvements in self-reported staff confidence and job satisfaction (Murray et al. 2019). As much of the harm that can be derived from the label attached to care rests upon the beliefs and attitudes underpinning that label, it is crucial that hospital staff understand the impacts of different types and stages of dementia. Embedding high quality training^1^ that emphasizes the relational aspects of care may promote the dignity, autonomy, and self-worth of the person with dementia, and reduce the likelihood that the identifier is used as a proxy for a diagnostic label.

However, there are wider aspects of care related to the environment within which the visual identifier is embedded that are more difficult to control. In their ethnographic work, Featherstone and colleagues “found the use of such technologies to be nested within a context of wider cultural understandings” (Featherstone, Boddington, and Northcott 2020, 10). If visual identification systems are to be implemented effectively then further research is needed to examine what aspects of care are components of the visual identification system, and which are facets of the wider culture of care that operates in the hospital or ward, as it is sometimes difficult to draw a clear line between context and intervention.

## Strengths and Weaknesses

This paper is the first to consider the ethical and legal principles engaged through the use of visual identification systems, to discuss the tensions and trade-offs that may arise between these principles in context and to make initial suggestions about how they may be addressed. However, there is limited empirical evidence regarding the use of visual identifiers and the benefits and harms they generate for different stakeholders. Therefore, it is also important to note that the principles, and the questions that they pose, are not exhaustive, and must be interpreted in light of findings from subsequent research and the wider context of implementation.

Further studies could help to advance this field. While we argue that the consideration of the principles could lead to more effective and ethical use of visual identifiers in the United Kingdom, research is needed to confirm whether these principles are generalizable to other applications (such as visual impairment and hearing loss) and to other jurisdictions. In making these comparisons, it is important to recognize that there may be different drivers for introducing signage. For example, the legal basis for signage highlighting infectious disease risk and the need for carers to wear personal protective equipment is found in public health law focusing on health protection: whilst such signs could be disclosive of a patient’s disease state, this concern could be regarded as subservient to the requirement for an employer to protect their staff.

Qualitative studies (including ethnographies and observational studies) exploring patient and hospital staff’s experiences of visual identifiers could help to identify key challenges and opportunities and may provide insights into stakeholder views around issues such as whether consent is needed and in what form; the degree of training and information required for hospital staff to feel comfortable using identifiers; and what form of identifier (or combination of identifiers) strikes the right balance between informative and overly burdensome for staff or infringing on patient privacy.

Given the proliferation of signage on hospital wards, future empirical research could help with understanding how symbols compete with each other in busy wards and how this might impact the use of a visual identifier.

We also acknowledge that visual identifiers for dementia have been implemented across the world, and the opportunities and challenges related to their use extend beyond the U.K. context. However, we have limited our analysis in this paper to the United Kingdom, as interpreting and translating the principles requires consideration of the local legal system and policy landscape. This has enabled us to strengthen our analysis of the tensions and challenges encountered in the application of visual identifiers in the United Kingdom. Further investigation around the use of visual identifiers in other jurisdictions is needed, particularly in low-income, low-resource countries where there are likely to be different tensions and competing priorities, highlighting the importance of context in any assessment of this topic.

## Conclusion

This paper has identified some ethical principles to help enable the assessment of relevant implications in the design phase of a visual identification system for hospitalized patients with dementia, so that decisions about potential risks and benefits can be made explicitly and purposefully. We have further explored some examples of when the principles are engaged and the trade-offs that are likely to result. As we have highlighted, resolving these trade-offs is not always straightforward, and is largely a matter of judgement, which must take into account the context and purpose for which the visual identifier is being used. Adopting a framework such as person-centred care may also help with ethical decision-making, and suggests that visual identifiers should be adopted as part of a broader care approach encompassing complementary strategies to maximize the benefits of this tool.

## Data Availability

A data availability statement is not applicable as this research did not involve the generation or collection of relevant data.

## Footnotes

1 Some visual identification systems, such as The Butterfly Scheme, are fully integrated schemes that include training for all staff interacting with patients.
